# *N*-[(*E*)-4-(Methyl­sulfon­yl)benzyl­idene]-3-nitro­aniline

**DOI:** 10.1107/S1600536811031539

**Published:** 2011-08-11

**Authors:** Yue-Hu Chen, Rong-Bao Ge, Hua-Jian Liu, Shao-Song Qian

**Affiliations:** a School of Life Sciences, ShanDong University of Technology, ZiBo 255049, People’s Republic of China

## Abstract

In the title compound, C_14_H_12_N_2_O_4_S, the dihedral angle between the two aromatic rings is 35.65 (12)°. The crystal packing is stabilized by weak C—H⋯O hydrogen bonds and aromatic π–π ring stacking inter­actions [minimum ring centroid separation = 3.697 (3) Å].

## Related literature

For pharmacological applications of Schiff bases, see: Venugopal & Jayashree (2008 ▶); Villar *et al.* (2004 ▶); Wadher *et al.* (2009 ▶). For similar structures, see: Qian & Cui (2009 ▶); Qian & Liu (2010 ▶). For comparative bond lengths, see: Allen *et al.* (1987 ▶).
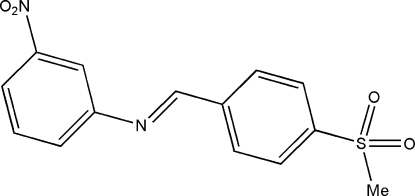


## Experimental

### 

#### Crystal data

C_14_H_12_N_2_O_4_S
*M* *_r_* = 304.32
Monoclinic, 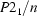

*a* = 12.707 (7) Å
*b* = 8.669 (5) Å
*c* = 14.257 (8) Å
β = 114.140 (5)°
*V* = 1433.2 (14) Å^3^
*Z* = 4
Mo *K*α radiation
μ = 0.24 mm^−1^
*T* = 296 K
0.25 × 0.23 × 0.21 mm

#### Data collection

Bruker APEXII CCD diffractometer
Absorption correction: multi-scan (*SADABS*; Bruker, 2004 ▶) *T*_min_ = 0.942, *T*_max_ = 0.951
9119 measured reflections
2525 independent reflections
1937 reflections with *I* > 2σ(*I*)
*R*_int_ = 0.026

#### Refinement

*R*[*F* ^2^ > 2σ(*F* ^2^)] = 0.037
*wR*(*F* ^2^) = 0.102
*S* = 1.02
2525 reflections
192 parameters
H-atom parameters constrained
Δρ_max_ = 0.28 e Å^−3^
Δρ_min_ = −0.21 e Å^−3^

### 

Data collection: *APEX2* (Bruker, 2004 ▶); cell refinement: *SAINT* (Bruker, 2004 ▶); data reduction: *SAINT*; program(s) used to solve structure: *SHELXS97* (Sheldrick, 2008 ▶); program(s) used to refine structure: *SHELXL97* (Sheldrick, 2008 ▶); molecular graphics: *SHELXTL* (Sheldrick, 2008 ▶); software used to prepare material for publication: *SHELXTL*.

## Supplementary Material

Crystal structure: contains datablock(s) global, I. DOI: 10.1107/S1600536811031539/zs2133sup1.cif

Structure factors: contains datablock(s) I. DOI: 10.1107/S1600536811031539/zs2133Isup2.hkl

Supplementary material file. DOI: 10.1107/S1600536811031539/zs2133Isup3.cml

Additional supplementary materials:  crystallographic information; 3D view; checkCIF report

## Figures and Tables

**Table 1:** Hydrogen-bond geometry (Å, °)

*D*—H⋯*A*	*D*—H	H⋯*A*	*D*⋯*A*	*D*—H⋯*A*
C14—H14*C*⋯O1^i^	0.96	2.42	3.380 (3)	178
C12—H12⋯O5^ii^	0.93	2.59	3.273 (4)	131
C6—H6⋯O2^iii^	0.93	2.52	3.442 (3)	169
C5—H5⋯O4^iv^	0.93	2.41	3.249 (3)	150

Symmetry codes: (i) 
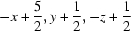
; (ii) 
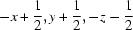
; (iii) 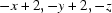; (iv) 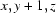.

**Fig. 1.**
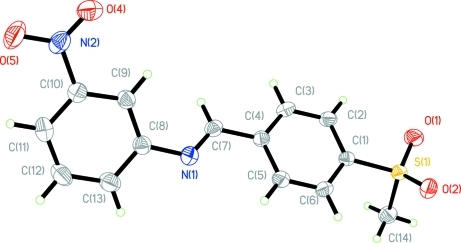
The structure of the title compound, showing the atom-labelling scheme. Displacement ellipsoids are drawn at the 30% probability level.

**Fig. 2.**
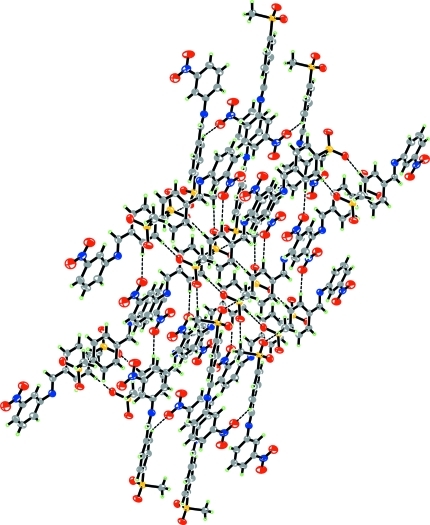
Hydrogen-bonding interactions in the title compound, shown as dashed lines.

## supplementary crystallographic information

### Comment

Schiff base compounds have been of great interest owing to their wide range of biological activities. They have been found to possess pharmacological activities, such as anti-cancer (Villar *et al.*, 2004), anti-bacterial (Venugopal & Jayashree, 2008), anti-inflammatory, anti-microbial and anti-viral (Wadher *et al.*, 2009). As an extension of our work on the structural characterization of Schiff base compounds, the crystal structure of the title compound C_14_H_12_N_2_O_4_S is reported here. In this compound (Fig. 1), all bond lengths are within normal ranges (Allen *et al.*, 1987) and comparable with the values observed in two closely related compounds (Qian & Cui, 2009; Qian & Liu, 2010). The dihedral angle between the two aromatic rings is 35.65 (12)°. The crystal packing is stabilized by weak C—H···O hydrogen bonds (Table 1) and aromatic π-π stacking interactions [minimum ring centroid–centroid distance, 3.697 (3)Å] (Fig. 2).

### Experimental

4-(Methylsulfonyl)benzaldehyde (0.184 g) and 2,6-diisopropylaniline (0.138 g) were dissolved in acetonitrile (20 ml). The mixture was stirred at room temperature for 10 min to give a clear yellow solution. After allowing the solution to evaporate in air for 5 days, yellow block-shaped crystals of the title compound were obtained.

### Refinement

All H atoms were placed in geometrical positions and constrained to ride on their parent atoms with C—H distances in the range 0.93–0.96 Å. These were treated as riding atoms, with *U*_iso_(H) = k*U*_eq_(C), where k = 1.5 for methyl and 1.2 for all other H atoms.

### Crystal data

**Table:**

C_14_H_12_N_2_O_4_S	*F*(000) = 632
*M**_r_* = 304.32	*D*_x_ = 1.410 Mg m^−^^3^
Monoclinic, *P*2_1_/*n*	Mo *K*α radiation, λ = 0.71073 Å
Hall symbol: -P 2yn	Cell parameters from 3721 reflections
*a* = 12.707 (7) Å	θ = 2.8–26.0°
*b* = 8.669 (5) Å	µ = 0.24 mm^−^^1^
*c* = 14.257 (8) Å	*T* = 296 K
β = 114.140 (5)°	Block, yellow
*V* = 1433.2 (14) Å^3^	0.25 × 0.23 × 0.21 mm
*Z* = 4	

### Data collection

**Table:**

Bruker APEXII CCD diffractometer	2525 independent reflections
Radiation source: fine-focus sealed tube	1937 reflections with *I* > 2σ(*I*)
graphite	*R*_int_ = 0.026
φ and ω scans	θ_max_ = 25.0°, θ_min_ = 2.8°
Absorption correction: multi-scan (*SADABS*; Bruker, 2004)	*h* = −15→15
*T*_min_ = 0.942, *T*_max_ = 0.951	*k* = −10→9
9119 measured reflections	*l* = −16→16

### Refinement

**Table:**

Refinement on *F*^2^	Secondary atom site location: difference Fourier map
Least-squares matrix: full	Hydrogen site location: inferred from neighbouring sites
*R*[*F*^2^ > 2σ(*F*^2^)] = 0.037	H-atom parameters constrained
*wR*(*F*^2^) = 0.102	*w* = 1/[σ^2^(*F*_o_^2^) + (0.0358*P*)^2^ + 0.9892*P*] where *P* = (*F*_o_^2^ + 2*F*_c_^2^)/3
*S* = 1.02	(Δ/σ)_max_ < 0.001
2525 reflections	Δρ_max_ = 0.28 e Å^−^^3^
192 parameters	Δρ_min_ = −0.21 e Å^−^^3^
0 restraints	Extinction correction: *SHELXL97* (Sheldrick, 2008), Fc^*^=kFc[1+0.001xFc^2^λ^3^/sin(2θ)]^-1/4^
Primary atom site location: structure-invariant direct methods	Extinction coefficient: 0.0103 (10)

### Special details

**Table:**

Geometry. All esds (except the esd in the dihedral angle between two l.s. planes) are estimated using the full covariance matrix. The cell esds are taken into account individually in the estimation of esds in distances, angles and torsion angles; correlations between esds in cell parameters are only used when they are defined by crystal symmetry. An approximate (isotropic) treatment of cell esds is used for estimating esds involving l.s. planes.
Refinement. Refinement of F^2^ against ALL reflections. The weighted R-factor wR and goodness of fit S are based on F^2^, conventional R-factors R are based on F, with F set to zero for negative F^2^. The threshold expression of F^2^ > 2sigma(F^2^) is used only for calculating R-factors(gt) etc. and is not relevant to the choice of reflections for refinement. R-factors based on F^2^ are statistically about twice as large as those based on F, and R- factors based on ALL data will be even larger.

### Fractional atomic coordinates and isotropic or equivalent isotropic displacement parameters (Å^2^)

**Table:**

	*x*	*y*	*z*	*U*_iso_*/*U*_eq_	
C1	1.01452 (18)	0.6909 (2)	0.10542 (17)	0.0405 (5)	
C2	1.0352 (2)	0.5465 (3)	0.15075 (18)	0.0475 (6)	
H2	1.1079	0.5208	0.2000	0.057*	
C3	0.9452 (2)	0.4404 (3)	0.12122 (18)	0.0498 (6)	
H3	0.9580	0.3433	0.1515	0.060*	
C4	0.83654 (19)	0.4776 (3)	0.04718 (17)	0.0420 (5)	
C5	0.8187 (2)	0.6221 (3)	0.00106 (19)	0.0507 (6)	
H5	0.7467	0.6471	−0.0496	0.061*	
C6	0.90665 (19)	0.7287 (3)	0.02969 (18)	0.0500 (6)	
H6	0.8941	0.8253	−0.0013	0.060*	
C7	0.7403 (2)	0.3652 (3)	0.01717 (18)	0.0479 (6)	
H7	0.7518	0.2703	0.0503	0.058*	
C8	0.5512 (2)	0.2885 (3)	−0.08472 (18)	0.0496 (6)	
C9	0.5682 (2)	0.1309 (3)	−0.09320 (17)	0.0485 (6)	
H9	0.6417	0.0912	−0.0765	0.058*	
C10	0.4727 (2)	0.0354 (3)	−0.12720 (17)	0.0504 (6)	
C11	0.3618 (2)	0.0891 (4)	−0.1524 (2)	0.0648 (8)	
H11	0.2994	0.0217	−0.1736	0.078*	
C12	0.3465 (2)	0.2452 (4)	−0.1451 (2)	0.0703 (8)	
H12	0.2728	0.2841	−0.1621	0.084*	
C13	0.4393 (2)	0.3445 (3)	−0.1129 (2)	0.0640 (7)	
H13	0.4273	0.4499	−0.1099	0.077*	
N1	0.64205 (17)	0.3974 (2)	−0.05349 (16)	0.0530 (5)	
N2	0.4908 (2)	−0.1309 (3)	−0.13755 (16)	0.0634 (6)	
O1	1.22903 (14)	0.7666 (2)	0.21956 (15)	0.0694 (6)	
O2	1.13137 (15)	0.8930 (2)	0.05065 (14)	0.0609 (5)	
C14	1.0752 (2)	0.9783 (3)	0.1990 (2)	0.0600 (7)	
H14A	1.0613	0.9364	0.2552	0.090*	
H14B	1.0048	1.0201	0.1486	0.090*	
H14C	1.1320	1.0585	0.2238	0.090*	
O4	0.5876 (2)	−0.1747 (2)	−0.12006 (19)	0.0886 (7)	
O5	0.4073 (2)	−0.2168 (3)	−0.1646 (2)	0.1008 (8)	
S1	1.12545 (5)	0.83166 (7)	0.14267 (5)	0.0477 (2)	

### Atomic displacement parameters (Å^2^)

**Table:**

	*U*^11^	*U*^22^	*U*^33^	*U*^12^	*U*^13^	*U*^23^
C1	0.0409 (11)	0.0355 (12)	0.0461 (12)	0.0032 (10)	0.0189 (10)	−0.0023 (10)
C2	0.0451 (13)	0.0397 (13)	0.0495 (13)	0.0046 (11)	0.0109 (10)	0.0002 (11)
C3	0.0609 (15)	0.0332 (12)	0.0526 (14)	0.0031 (11)	0.0205 (12)	0.0031 (10)
C4	0.0447 (12)	0.0378 (13)	0.0455 (12)	−0.0008 (10)	0.0204 (10)	−0.0055 (10)
C5	0.0425 (13)	0.0461 (14)	0.0567 (14)	0.0027 (11)	0.0132 (11)	0.0048 (11)
C6	0.0482 (13)	0.0381 (13)	0.0581 (15)	0.0026 (11)	0.0161 (11)	0.0090 (11)
C7	0.0551 (14)	0.0400 (13)	0.0518 (14)	−0.0005 (11)	0.0250 (12)	−0.0037 (11)
C8	0.0471 (13)	0.0506 (15)	0.0488 (14)	−0.0002 (11)	0.0174 (11)	−0.0008 (11)
C9	0.0424 (12)	0.0519 (15)	0.0475 (13)	0.0001 (11)	0.0147 (10)	0.0013 (11)
C10	0.0514 (14)	0.0535 (15)	0.0433 (13)	−0.0007 (12)	0.0161 (11)	0.0014 (11)
C11	0.0479 (15)	0.081 (2)	0.0619 (17)	−0.0114 (14)	0.0186 (12)	−0.0038 (15)
C12	0.0443 (14)	0.084 (2)	0.0773 (19)	0.0097 (14)	0.0189 (13)	−0.0106 (17)
C13	0.0541 (15)	0.0622 (17)	0.0695 (17)	0.0086 (13)	0.0191 (13)	−0.0076 (14)
N1	0.0483 (12)	0.0473 (12)	0.0613 (13)	−0.0043 (10)	0.0202 (10)	−0.0040 (10)
N2	0.0696 (15)	0.0529 (14)	0.0540 (13)	−0.0085 (13)	0.0114 (11)	0.0022 (11)
O1	0.0432 (9)	0.0555 (11)	0.0905 (14)	0.0030 (8)	0.0081 (9)	0.0054 (10)
O2	0.0639 (11)	0.0529 (11)	0.0763 (12)	−0.0058 (9)	0.0392 (10)	0.0029 (9)
C14	0.0634 (16)	0.0438 (15)	0.0705 (17)	−0.0047 (12)	0.0249 (14)	−0.0131 (13)
O4	0.0781 (15)	0.0586 (13)	0.1134 (18)	0.0129 (11)	0.0232 (13)	0.0019 (12)
O5	0.0914 (16)	0.0647 (14)	0.1174 (19)	−0.0310 (13)	0.0134 (14)	−0.0079 (13)
S1	0.0405 (3)	0.0382 (3)	0.0612 (4)	0.0001 (3)	0.0177 (3)	−0.0009 (3)

### Geometric parameters (Å, °)

**Table:**

C1—C2	1.385 (3)	C9—C10	1.383 (3)
C1—C6	1.393 (3)	C9—H9	0.9300
C1—S1	1.773 (2)	C10—C11	1.384 (4)
C2—C3	1.391 (3)	C10—N2	1.477 (3)
C2—H2	0.9300	C11—C12	1.377 (4)
C3—C4	1.391 (3)	C11—H11	0.9300
C3—H3	0.9300	C12—C13	1.378 (4)
C4—C5	1.390 (3)	C12—H12	0.9300
C4—C7	1.483 (3)	C13—H13	0.9300
C5—C6	1.377 (3)	N2—O4	1.212 (3)
C5—H5	0.9300	N2—O5	1.222 (3)
C6—H6	0.9300	O1—S1	1.4394 (18)
C7—N1	1.274 (3)	O2—S1	1.446 (2)
C7—H7	0.9300	C14—S1	1.757 (3)
C8—C9	1.396 (4)	C14—H14A	0.9600
C8—C13	1.398 (3)	C14—H14B	0.9600
C8—N1	1.415 (3)	C14—H14C	0.9600
			
C2—C1—C6	120.9 (2)	C9—C10—N2	117.9 (2)
C2—C1—S1	120.43 (17)	C11—C10—N2	119.1 (2)
C6—C1—S1	118.68 (17)	C12—C11—C10	118.1 (3)
C1—C2—C3	118.7 (2)	C12—C11—H11	121.0
C1—C2—H2	120.6	C10—C11—H11	121.0
C3—C2—H2	120.6	C11—C12—C13	120.8 (3)
C4—C3—C2	121.0 (2)	C11—C12—H12	119.6
C4—C3—H3	119.5	C13—C12—H12	119.6
C2—C3—H3	119.5	C12—C13—C8	120.7 (3)
C5—C4—C3	119.2 (2)	C12—C13—H13	119.7
C5—C4—C7	120.0 (2)	C8—C13—H13	119.7
C3—C4—C7	120.9 (2)	C7—N1—C8	120.8 (2)
C6—C5—C4	120.6 (2)	O4—N2—O5	123.4 (3)
C6—C5—H5	119.7	O4—N2—C10	118.2 (2)
C4—C5—H5	119.7	O5—N2—C10	118.4 (3)
C5—C6—C1	119.6 (2)	S1—C14—H14A	109.5
C5—C6—H6	120.2	S1—C14—H14B	109.5
C1—C6—H6	120.2	H14A—C14—H14B	109.5
N1—C7—C4	120.7 (2)	S1—C14—H14C	109.5
N1—C7—H7	119.7	H14A—C14—H14C	109.5
C4—C7—H7	119.7	H14B—C14—H14C	109.5
C9—C8—C13	119.4 (2)	O1—S1—O2	117.69 (12)
C9—C8—N1	123.0 (2)	O1—S1—C14	109.00 (13)
C13—C8—N1	117.5 (2)	O2—S1—C14	108.15 (13)
C10—C9—C8	118.1 (2)	O1—S1—C1	109.01 (11)
C10—C9—H9	120.9	O2—S1—C1	108.14 (11)
C8—C9—H9	120.9	C14—S1—C1	103.97 (12)
C9—C10—C11	123.0 (3)		
			
C6—C1—C2—C3	−1.7 (4)	C10—C11—C12—C13	−0.5 (4)
S1—C1—C2—C3	178.49 (18)	C11—C12—C13—C8	−1.4 (5)
C1—C2—C3—C4	0.4 (4)	C9—C8—C13—C12	2.5 (4)
C2—C3—C4—C5	1.1 (4)	N1—C8—C13—C12	179.3 (2)
C2—C3—C4—C7	−179.0 (2)	C4—C7—N1—C8	177.8 (2)
C3—C4—C5—C6	−1.4 (4)	C9—C8—N1—C7	−38.7 (4)
C7—C4—C5—C6	178.7 (2)	C13—C8—N1—C7	144.5 (2)
C4—C5—C6—C1	0.1 (4)	C9—C10—N2—O4	−3.1 (4)
C2—C1—C6—C5	1.4 (4)	C11—C10—N2—O4	176.4 (2)
S1—C1—C6—C5	−178.75 (19)	C9—C10—N2—O5	177.9 (2)
C5—C4—C7—N1	2.7 (3)	C11—C10—N2—O5	−2.6 (4)
C3—C4—C7—N1	−177.3 (2)	C2—C1—S1—O1	1.1 (2)
C13—C8—C9—C10	−1.5 (4)	C6—C1—S1—O1	−178.74 (19)
N1—C8—C9—C10	−178.2 (2)	C2—C1—S1—O2	130.2 (2)
C8—C9—C10—C11	−0.4 (4)	C6—C1—S1—O2	−49.7 (2)
C8—C9—C10—N2	179.1 (2)	C2—C1—S1—C14	−115.1 (2)
C9—C10—C11—C12	1.4 (4)	C6—C1—S1—C14	65.1 (2)
N2—C10—C11—C12	−178.1 (2)		

### Hydrogen-bond geometry (Å, °)

**Table:**

*D*—H···*A*	*D*—H	H···*A*	*D*···*A*	*D*—H···*A*
C14—H14C···O1^i^	0.96	2.42	3.380 (3)	178
C12—H12···O5^ii^	0.93	2.59	3.273 (4)	131
C6—H6···O2^iii^	0.93	2.52	3.442 (3)	169
C5—H5···O4^iv^	0.93	2.41	3.249 (3)	150
C2—H2···O1	0.93	2.58	2.948 (3)	104

Symmetry codes: (i) −*x*+5/2, *y*+1/2, −*z*+1/2; (ii) −*x*+1/2, *y*+1/2, −*z*−1/2; (iii) −*x*+2, −*y*+2, −*z*; (iv) *x*, *y*+1, *z*.
